# Taphonomy and palaeoecology of Late Triassic (Carnian) ammonoid concentrations from the Taurus Mountains, Turkey

**DOI:** 10.1111/let.12179

**Published:** 2016-05-21

**Authors:** Susanne Mayrhofer, Alexander Lukeneder, Leopold Krystyn

**Affiliations:** ^1^ Department for Exhibition and EducationNatural History Museum ViennaBurgring 71010ViennaAustria; ^2^ Geological‐Paleontological DepartmentNatural History Museum ViennaBurgring 71010ViennaAustria; ^3^ Department of PalaeontologyUniversity of ViennaAlthanstraße 141010ViennaAustria

**Keywords:** Ammonoids, Carnian Crisis, *Kasimlarceltites*, Late Triassic, spatial shell orientation, taphonomy, Taurus Mountains, Turkey

## Abstract

The deposits of the Carnian Kasımlar Formation within the Taurus Platform Units of south‐western Turkey represent an important archive of a Late Triassic ecosystem. New palaeontological information was obtained by analysing the *Kasimlarceltites* mass occurrence, located within the Kasımlar Formation and named after the Lower Carnian (Julian) ammonoid genus *Kasimlarceltites*. This is the dominant taxon (> 94%) within the mass occurrence: nearly 775 million ammonoids and 50 million gastropods were extrapolated for the whole extension (at least 5 km^2^) of the *Kasimlarceltites* beds. This calculation is one of the main findings within this study, as it is the first time that such a fossil mass occurrence was quantified. Additionally, orientation measurements of the planispiral ammonoids and the helical gastropods enabled reconstructing the history of the mass occurrence and interpreting the underlying transport mechanisms. Further taphonomic aspects (e.g. biofabric, preservation, bioerosion or genetic classification) as well as comparisons with samples of the same acme zone from different localities near Aşağiyaylabel (AS IV, KA I‐II) point to a two‐phased genetic history. Accordingly, local mass mortality within the *Kasimlarceltites* fauna due to oxygen fluctuations or methane degassing may have initially led to a primary accumulation. These deposits were then reworked and redeposited basinward by gravity flows to create the present‐day secondary allochthonous concentrations.

The interpretation of depositional conditions of ammonoid mass occurrences based on combined taphonomic and palaeoecological information has become an increasingly hot topic during the last few decades (e.g. Seilacher 1960, 1968, 1971, 1990; Maeda & Seilacher 1996; Keupp 1997; Maeda 1999; Lukeneder 2003, 2004, 2005; Fernández‐López 2007; Olivero 2007; Wani 2007; Reyment 2008; Tomašových & Schlögl 2008; Maeda & Shigeta 2009; Lukeneder & Mayrhofer 2014). The mass occurrence investigated here consists to 94–99% of the single species *Kasimlarceltites krystyni*, which is presently known only from the area around Aşağiyaylabel. The *Kasimlarceltites* mass occurrence was deposited during Carnian time (Late Triassic) within an intra‐shelf basin of the western Cimmerian terranes. The Carnian is best known for a Tethyan‐wide carbonate platform demise (Carnian Crisis = Carnian Pluvial Event; Simms & Ruffell 1989; Lukeneder *et al*. 2012). The deposition of these ammonoids at an equatorial position (for Turkey about 9° according to Stampfli *et al*. 2002 and Moix *et al*. 2008) makes them suitable for taphonomic investigations on the genesis of this huge shell deposit, but also yields new insights into the Carnian Crisis at equatorial regions. We therefore investigated and analysed parameters related to the orientation of the ammonoid shells. We adopted a new study‐approach on such planispirally coiled ammonoids, already tested by Lukeneder *et al*. (2014).

The study was performed to test the number and orientation of the ammonoids within the known extension of the *Kasimlarceltites* mass occurrence, and therefore represents a quantification of an ammonoid mass occurrence done for the first time. The quantitative data were interpreted in combination with qualitative information about the depositional conditions from different slices and thin‐sections taken from various parts of the mass occurrence. The main aim of this study was to solve the following research questions: Are these ammonoids orientated in the same way as already tested within the smaller reference block (done by Lukeneder *et al*. 2014), and what does this result mean for the depositional conditions? Does the orientation of the ammonoids fit with the taphonomic interpretation of this shell bed? How might the life habitat of *Kasimlarceltites* and the environmental conditions, which occurred throughout the deposition of this mass occurrence, looked like?

The answers to these questions shed light into the genesis of this mass occurrence and consequently into the environmental conditions which occurred throughout its deposition during the time of the Carnian Crisis.

## Geographical and geological setting

**Figure 1:**
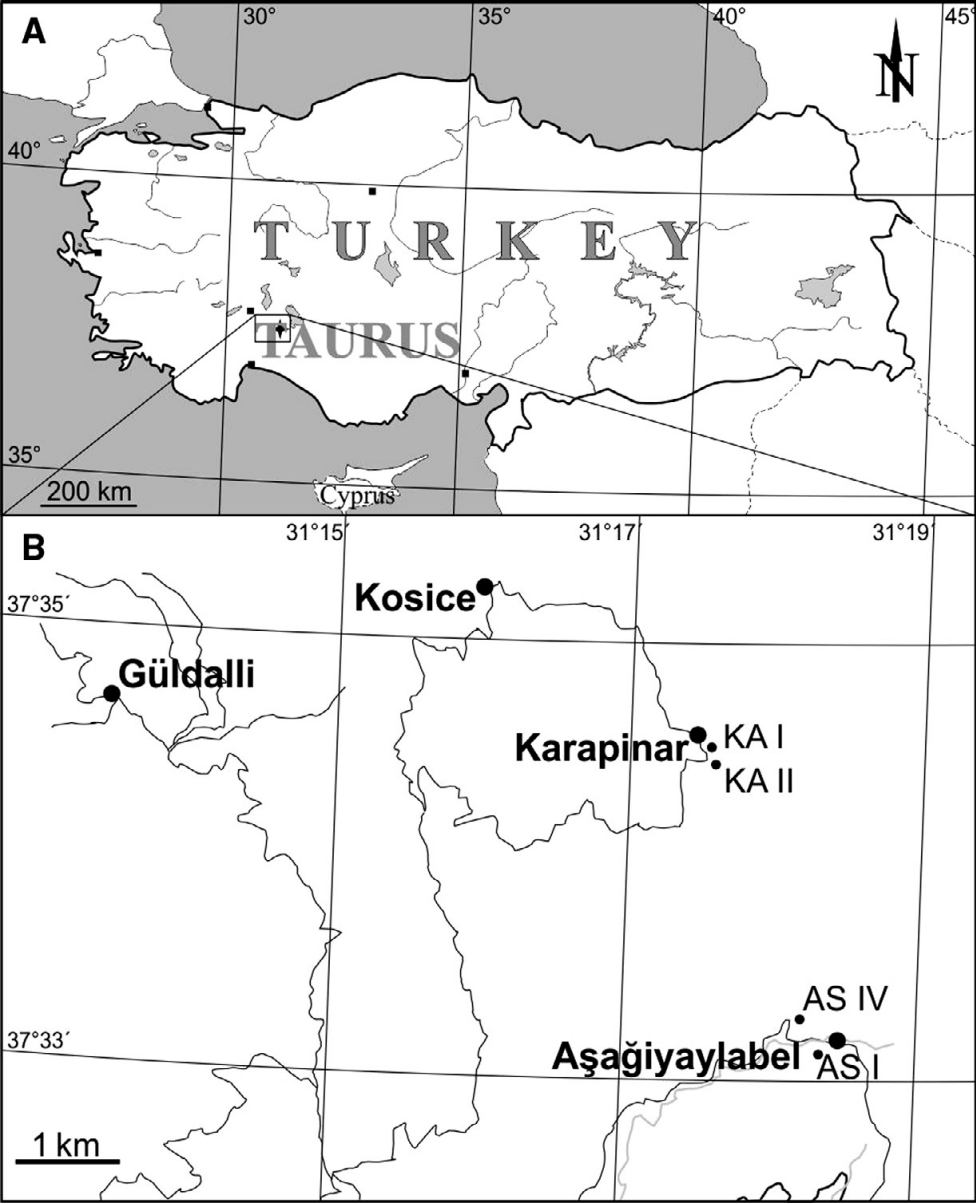
A, map of Turkey indicating the outcrop position of Aşağiyaylabel. B, detailed map of the area around Aşağiyaylabel and Karapinar.

The outcrop with the investigated bed AS 6 is situated near the village Aşağiyaylabel (Anatolia; N 37.551389° and E 31.304444°) in the western Taurus Mountains of Turkey (Fig. 1A, B). The sedimentary record at Aşağiyaylabel belongs to the top of the Kartoz and the base of the Kasımlar Formation and is therefore of late Early Carnian (Julian 2) to early Late Carnian age (Tuvalian 1). The Kasımlar Formation at Aşağiyaylabel represents one of the rare complete successions with a Lower to Upper Carnian ammonoid fauna (see Lukeneder & Lukeneder 2014).

**Figure 2:**
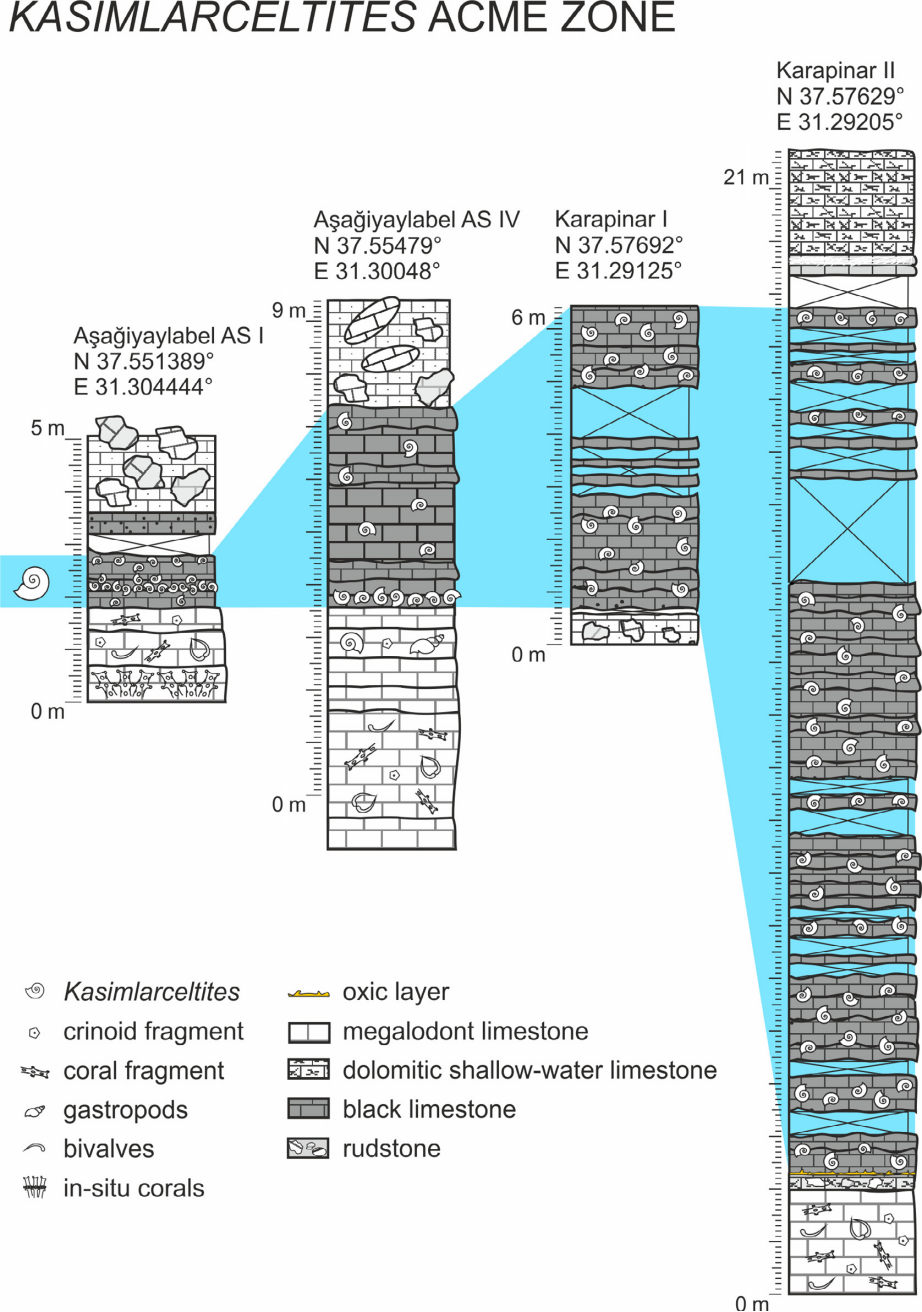
Logs of Aşağiyaylabel (AS I, AS IV) and Karapinar (KA I, KA II) with indicated position of the *Kasimlarceltites* acme zone (blue area).

The ammonoid coquinas (mass occurrence), on which this study focuses, belong to the Carbonate member (Unit A), near the bottom of the Kasımlar Formation. The *Kasimlarceltites* shell beds are composed of at least eight layers (1.8 m at AS I) to a maximum of 113 layers (16.5 m at KA II), with an extension over an area of at least 5 km^2^. Single beds are 2–40 cm thick. Detailed investigations were conducted in bed AS 6 of the section AS I, and compared with further samples from the *Kasimlarceltites* acme zone of the sections AS IV, KA I and KA II (Fig. 2). The layers of section AS IV are preserved in an overturned position. This is proven by geopetal fills, exhibiting their calcitic crystallisation (i.e. filling of voids, sediment free) at the ‘bottom’ of the ammonoids, as well as by the inverse gradation of the sediment. In addition, in the field, at section AS IV and sections KA I and KA II, the *Kasimlarceltites* beds were found above the rudstone layers and below the shallow‐water limestones in a stratigraphically overturned position. For a better comparison of the four localities, the stratigraphical overturn was corrected in the figured logs (Fig. 2).

## Material and methods

**Figure 3:**
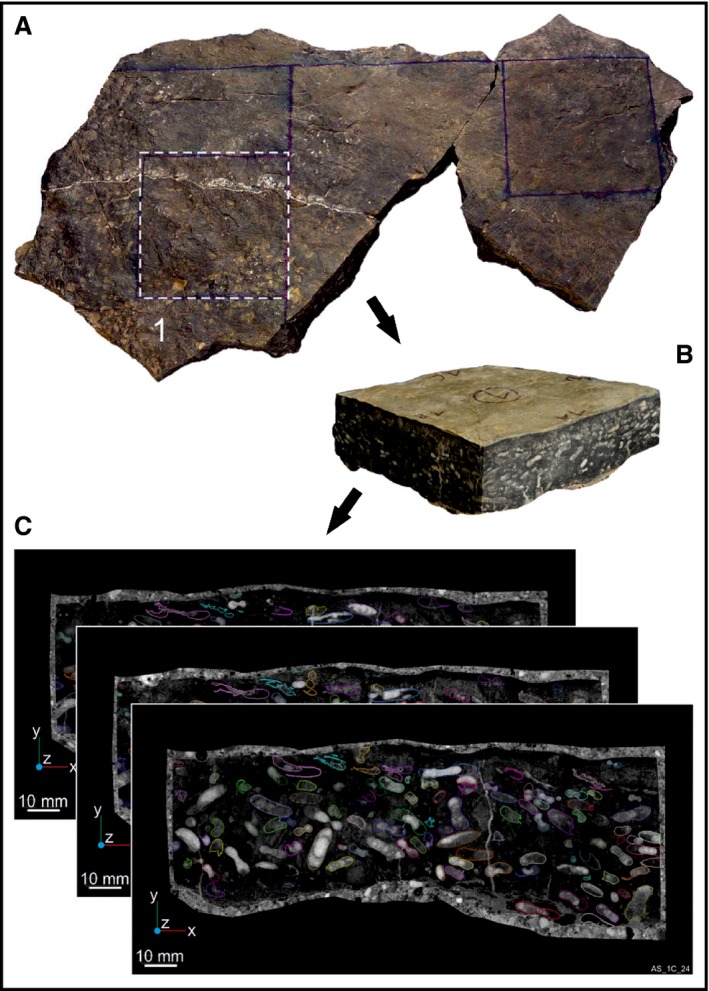
Steps from bed to ammonoid segmentation. A, ammonoid bed taken from the field (AS I, bed 6) with indicated position of the reference block. B, segmented reference block (150 × 45 × 140 mm). C, scanned slices of the reference block.

**Figure 4:**
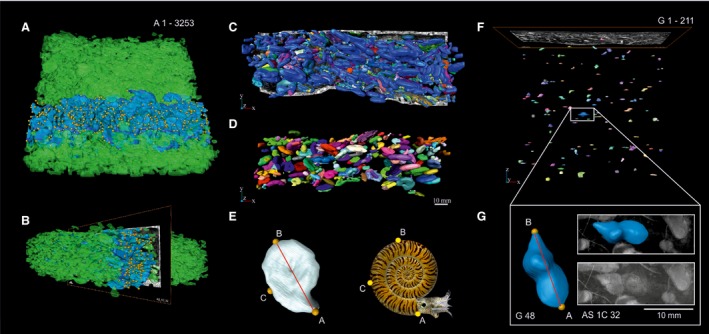
Ammonoid segmentation pictures (made with Amira). Virtual reconstruction and land‐mark‐setting of the ammonoids and gastropods from the reference block. A, B, 3,253 segmented ammonoids (blue and green ammonoids). Blue ammonoids (*n* = 675) already investigated in Lukeneder *et al*. (2014). A, view from top. B, side view. C, D, frontal view of segmented ammonoids. E, landmark positions (A–C), placed on every ammonoid surface and shown at a virtual ammonoid model designed by 7Reasons. F, segmented gastropods from the whole reference block. G, segmented gastropod with indicated landmarks (A, B) that were placed on every segmented gastropod.

The number and orientation of the ammonoids was tested based on a 150 × 45 × 140 mm reference block taken at the locality AS I from bed AS 6 (Fig. 3A). The block was cut from the limestone bed, ground and scanned at 2 mm intervals to produce 70 slices (Fig. 3A–C). From there the ammonoids were virtually segmented and a 3D‐surface rendered (Fig. 4A–E). The destructive method of grinding (Lukeneder *et al*. 2014; Naglik *et al*. 2015a,b; Tajika *et al*. 2015) was used because the non‐destructive method of computed tomography did not work due to similar density of the ammonoids (i.e. secondary calcite shells, about 2.6–2.8 g/cm^3^) and the host rock (limestone matrix, about 2.8 g/cm^3^).

**Figure 5:**
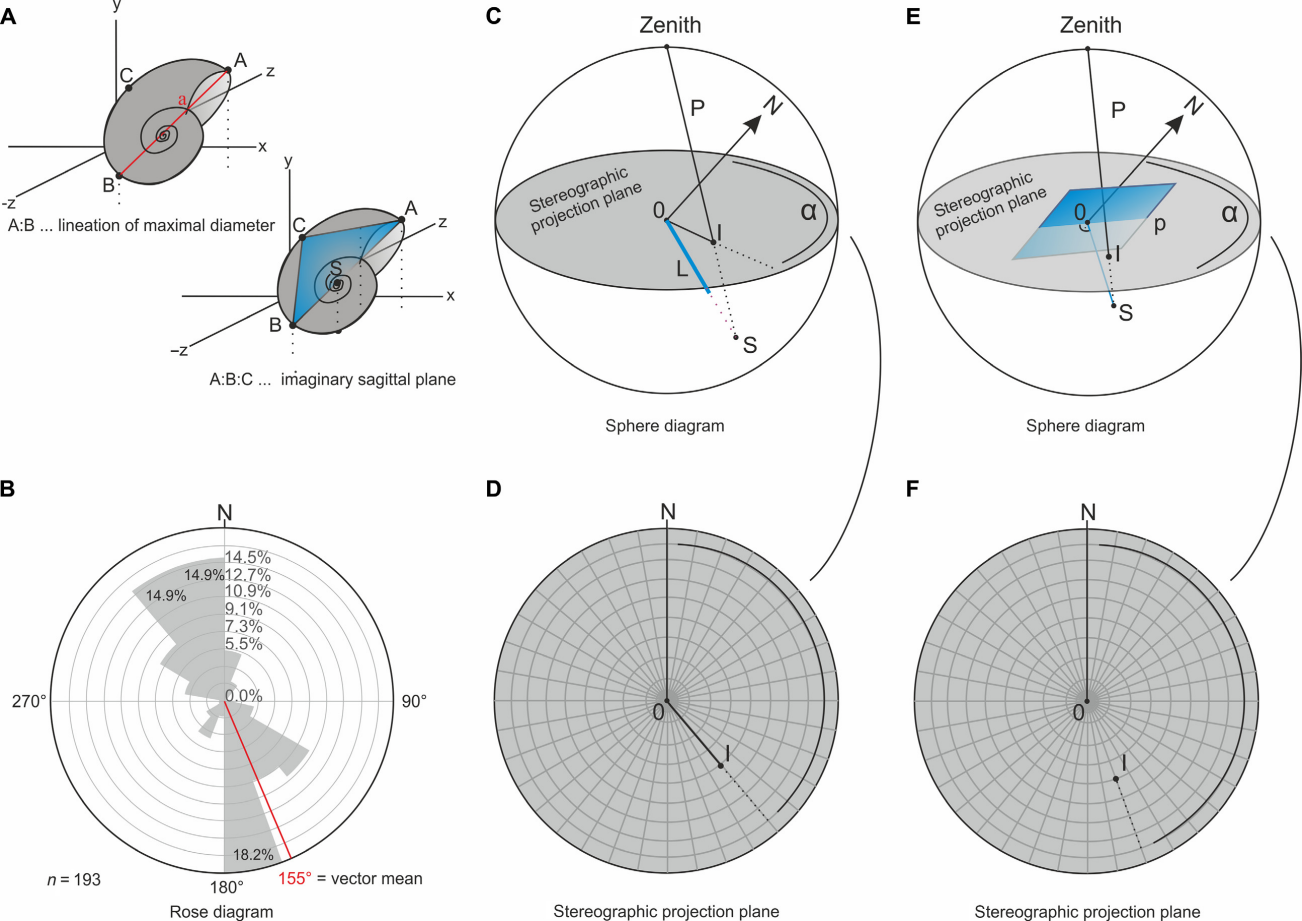
A, explanation of the orientation parameters used for subsequent investigation of the spatial orientation of each ammonoid. B, example of a rose diagram. C, sphere diagram with an intended lineation ‘L’ and its projection point ‘I’ at the stereographic projection plane. D, stereographic projection plane of the sphere diagram (C) at which the projection point ‘I’ of the lineation ‘L’ plots. E, sphere diagram with intended plane ‘p’ and its projection point ‘I’ at the stereographic projection plane. F, stereographic projection plane of the stereographic plot (E) with indicated projection point ‘I’ from the intended plane ‘p’.

The 3D‐visualization‐method described by Lukeneder *et al*. (2014) was used to analyse a possible spatial orientation of the ammonoids within the shell bed AS 6. The whole ammonoids, but also the gastropods (Fig. 4F, G), from the reference block were virtually segmented (Fig. 4A–G), counted and, if possible, their orientation analysed. By using landmark data (each segmented ammonoid was equipped with three and each segmented gastropod with two specified landmarks; Fig. 4E, G) the statistical orientation of each ammonoid and gastropod was investigated. Orientation parameters (maximum diameter, dip, dip direction and aperture direction of the lineation A:B through the gastropods and ammonoids, as well as dip and azimuth of an imaginary sagittal‐plane A‐B‐C through the ammonoids), already known from geological orientation measurements, were calculated (Fig. 5A).

Following Lukeneder *et al*. (2014) we used only those ammonoids for orientation analyses that were reconstructed from at least six slices when analysing the lineation A:B. At least three slices were required when analysing the imaginary sagittal‐plane (Fig. 5A). We furthermore adapted the method described by Lukeneder *et al*. (2014) for the segmented gastropods. Here, the orientation of the lineation A:B was analysed for 20 gastropods (intersected at least twice). For a detailed description of the method used see Lukeneder *et al*. (2014).

### Statistical methods for analysing the orientation

The spatial orientation of the segmented ammonoids and gastropods was statistically analysed with the software package Fabric8, usually used for the graphical display and analysis of tectonic data. The orientations of the aperture directions, as well as of the strike data were analysed within rose diagrams (Fig. 5B). Dip and dip direction of the lineations (A:B) as well as dip and azimuth of the planes (A‐B‐C) were analysed by plotting them stereographically (Fig. 5C–F). The orientation of 675 specimens of these ammonoids have already been published by Lukeneder *et al*. (2014).

### Rose diagrams

**Figure 6:**
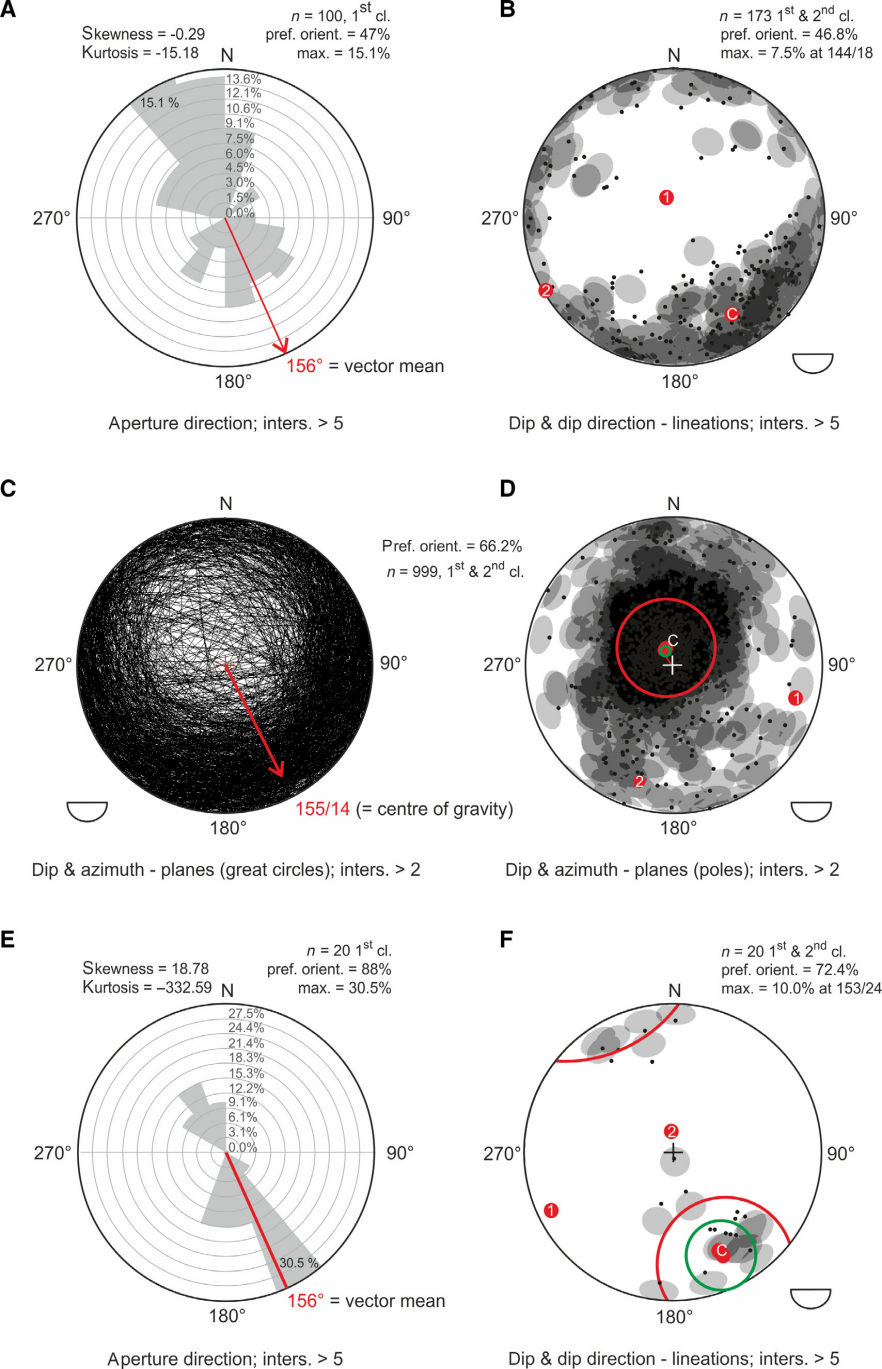
Results of analysed spatial shell orientation. A, rose diagram, indicating a bimodal SSE/NNW aperture orientation of the tested ammonoids with indicated aperture. B, dip and dip direction of the lineation (A:B) of the ammonoids, plotted on a stereographic projection plane. C, D, dip and azimuth of the planes (A‐B‐C), respectively ammonoids analysed within a sphere diagram. C, plot of great circles. D, plot of plane poles. E, rose diagram, indicating a bimodal SSE/NNW aperture orientation of the tested gastropods with indicated aperture. F, dip and dip direction of the lineation (A:B) of the gastropods, plotted on a stereographic projection plane.

Rose diagrams (Figs 5B, 6A, E) are circular histograms used for graphical analyses of pure directional data in a two‐dimensional way, without dip, dip direction, or strike. Raw data of directions were classified according to defined intervals. These intervals are drawn as sectors into a circle with a radius defined such that the most frequent percentage of the raw data directions plots at the circumference. Charting the percentages of the intervals into the diagram yields a graphical interpretation (Figs 5B, 6A, E). The software Fabric8 enabled statistical analyses of the directional data, complementing the graphical method. For more details about rose diagrams see Wallbrecher (1986).

### Stereographic plots

Stereographic plots (Figs 5C–F, 6B–D, F) were used to analyse spatial directional data in a three‐dimensional way. Pure directions were analysed in combination with their angle of dip (e.g. dip + dip direction of a lineation, respectively dip + azimuth of a plane). The stereographic projection (Fig. 5D, F) represents the equatorial plane of the so‐called ‘sphere diagram’ (Fig. 5C, E). When placing a projected lineation ‘L’ (Fig. 5C) within this sphere diagram, the intersection point of the elongation of this lineation ‘L’ with the lower hemisphere represents the sphere projection point ‘S’. When projecting a lineation from point ‘S’ to the zenith of this sphere diagram, the intersection point ‘I’ at the equatorial plane represents the data point ‘I’ of the lineation ‘L’ at the stereographic plot (Fig. 5C, D). The same procedure can be performed with single planes. When projecting a plane within this sphere diagram (Fig. 5E), the intersection point ‘I’ of the sphere projection point ‘S’ of an imaginary orthogonal lineation from the plane ‘p’ towards the sphere represents the data‐point ‘I’ of the plane ‘p’ on the stereographic plot (Fig. 5E, F). The stereographic projection plane bears a net of curves of constant latitudes and longitudes (Mardia & Jupp 2000). A more detailed description of stereographic plots and sphere diagrams is given by Wallbrecher (1978, 1979, 1986). Compared to other statistical methods of analysing directional data, the advantage of stereographic plots is the spatial approach. The possibility of representing planes and lineations in the stereographic plot with a single point allows dealing with large data‐sets (Adler *et al*. 1982; Wallbrecher 1986). The strike and azimuth of single points nevertheless can be precisely quantified. Complex statistical analyses are replaced by investigating maxima and fields of similar population density (Adler *et al*. 1982). The stereographic plot is mostly used for tectonic, but also for sedimentological analyses. For example, Potter & Pettijohn (1977) used stereographic plots to analyse disc‐shaped particles in respect to current versus gravity transport. Within our study, Potter & Pettijohn′s method is adapted for orientation analyses of planispiral cephalopods. Azimuth and azimuth direction of the ammonoids should be analysed in the same way as the disc‐shaped particles described by Potter & Pettijohn (1977).

## Results

As described by Kidwell *et al*. (1986), shell concentrations can be classified using a descriptive nomenclature (taxonomic composition, biofabric, geometry and internal structure) as well as a genetic classification (lithological‐, biological‐ or diagenetic genesis; see Martin 1999). To analyse the *Kasimlarceltites* mass occurrence from Aşağiyaylabel, we used Kidwell′s approaches. We also applied terminological terms of Seilacher (1990) to estimate the ancient environment of the depositional area and the mechanisms that led to the genesis of these fossil ammonoid concentrations.

### Geometry and internal structure

Following Kidwell *et al*. (1986) the mass occurrence has to be interpreted as a ‘bed’ instead of clump, pod, lens or wedge. The lateral distribution of the *Kasimlarceltites* layers differs at each section between 20 and 50 m: more than 30 m at AS I and 50 m at AS II and AS IV, as well as over 20 m at KA I and KA III and 50 m at KA II. Due to the known distribution, we assume that it represents a more or less consistent three‐dimensional structure over an area of about 5 km^2^ (Fig. 1). The whole acme zone, which consists of several beds (e.g. bed AS 6), is 1.8 m (at AS I) to 16.5 m thick (at KA II; Lukeneder & Mayrhofer 2014), and alternates between beds full of ammonoids and beds yielding only scattered ammonoid shell remains (Fig. 2). The accumulation beds generally show the same thickness and shell density along the whole extension. The internal structure of the *Kasimlarceltites* mass occurrence is not uniform, but shows an upward fining trend in bioclasts. The lower part of the mass occurrence at bed AS 6 consists of almost entire ammonoid shells, whereas the topmost third of the bed shows partly fragmented ammonoid shells which are topped by densely packed halobiid bivalve shell remains, and therefore represent a graded sediment structure.

### Lithology and microfacies of the host sediment

**Figure 7:**
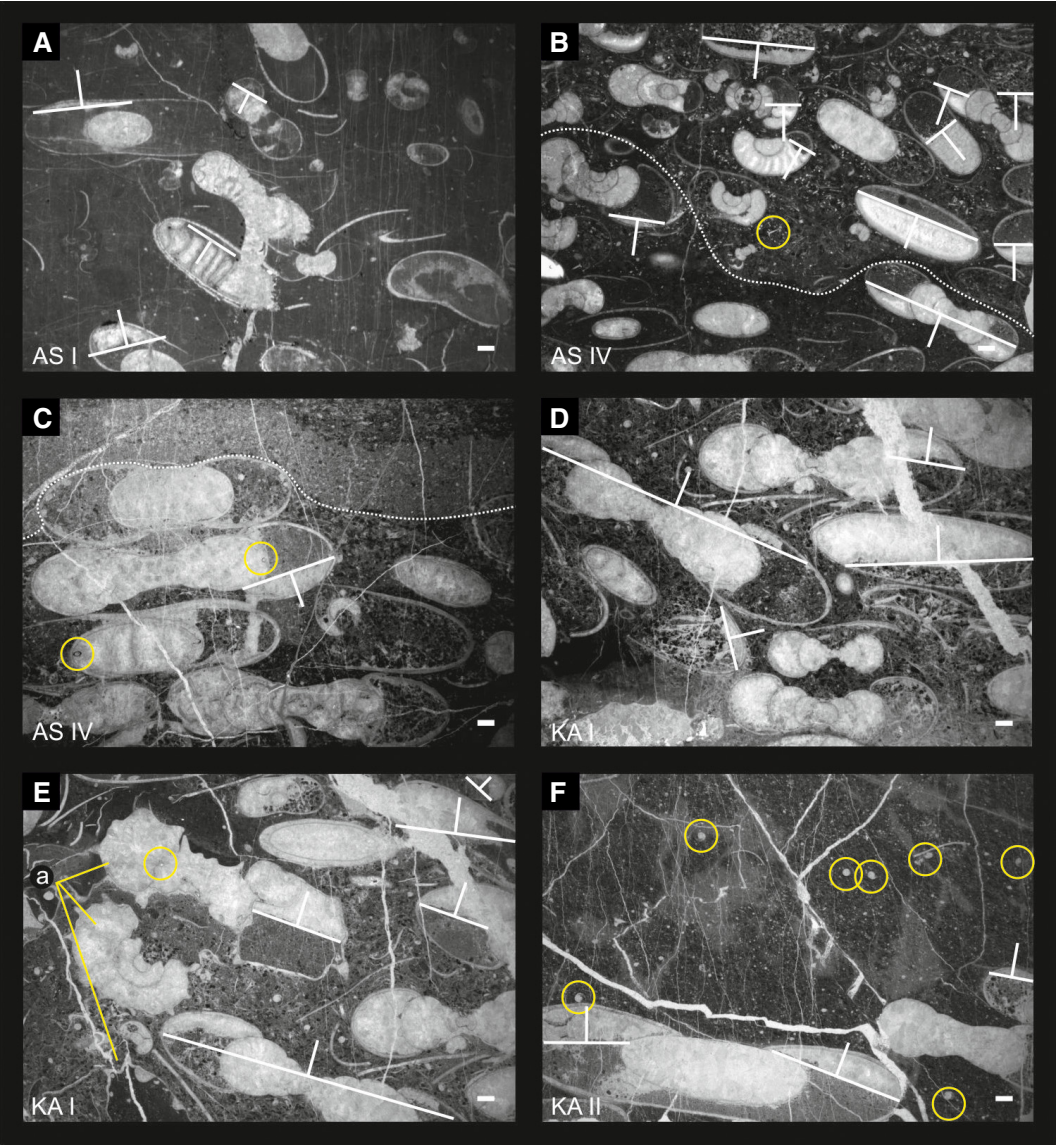
Thin‐sections of the *Kasimlarceltites* beds from different localities of the *Kasimlarceltites* acme zone. A, AS I, bed 6 – bioclastic pelagic floatstone. B, C, AS IV bed C – bioclastic packstone resp. floatstone with well‐preserved sponge spicules (B, yellow circle); note the geopetal fills, which indicates an inverse position of this layer/section, and the fine‐developed calcitic preservation of the phragmocones with their single chambers (B) as well as the siphonal tubes (C, yellow circle). C, NHMW 2014/0091/0008. D, E, KA I ‐ NHMW 2014/0092/0003 – bioclastic packstone resp. floatstone; in addition to the dominant *Kasimlarceltites* (D), additional ammonoid genera (E) are well preserved at the mass occurrence of KA I. F, KA II – NHMW2014/0092/0004 – pelagic bioclastic wackestone, small ammonoid shells (ammonitellae, juveniles or microconchs = yellow circles) are mixed with large shells (adult forms or macroconchs. All scale bars: 1 mm.

The background sediment of bed AS 6 represents a bioclastic pelagic wackestone (Fig. 7A), deposited within a deep shelf or mid‐ramp (*sensu* Flügel 2004). The thin‐shelled halobiid bivalves, deposited in masses at the top of the bed, generally occur in deep‐water, oxygen‐deficient settings or in ‘pelagic’ or ‘filamentous’ limestones, which represent fully oxygenated marine settings (McRoberts 2000, 2010; McRoberts *et al*. 2008; Lukeneder *et al*. 2012). Lagenid Foraminifera, *Omphaloptycha*‐like gastropods and some thick‐shelled bivalves, which also occur in bed AS 6 and within under‐ and overlying beds, represent allochthonous biogens, transported from the fore slope or shallow‐marine ramp (Lukeneder *et al*. 2012). Geopetal fills show different directions, pointing to a phase of reworking following pre‐lithification of the ammonoids (Fig. 7A, B). Compared to bed 6 of section AS I, the background sediment at the localities AS IV and KA I is more grain‐supported, hence representing a bioclastic packstone (Fig. 7B–E). This is in contrast to wackestone, which occurs both at section AS I (Fig. 7A) and section KA II (Fig. 7F). Anyhow, due to the high density of allochthonous biogens, at both localities, the fabric of the accumulation layers represent floatstones.

### Taxonomic composition

**Figure 8:**
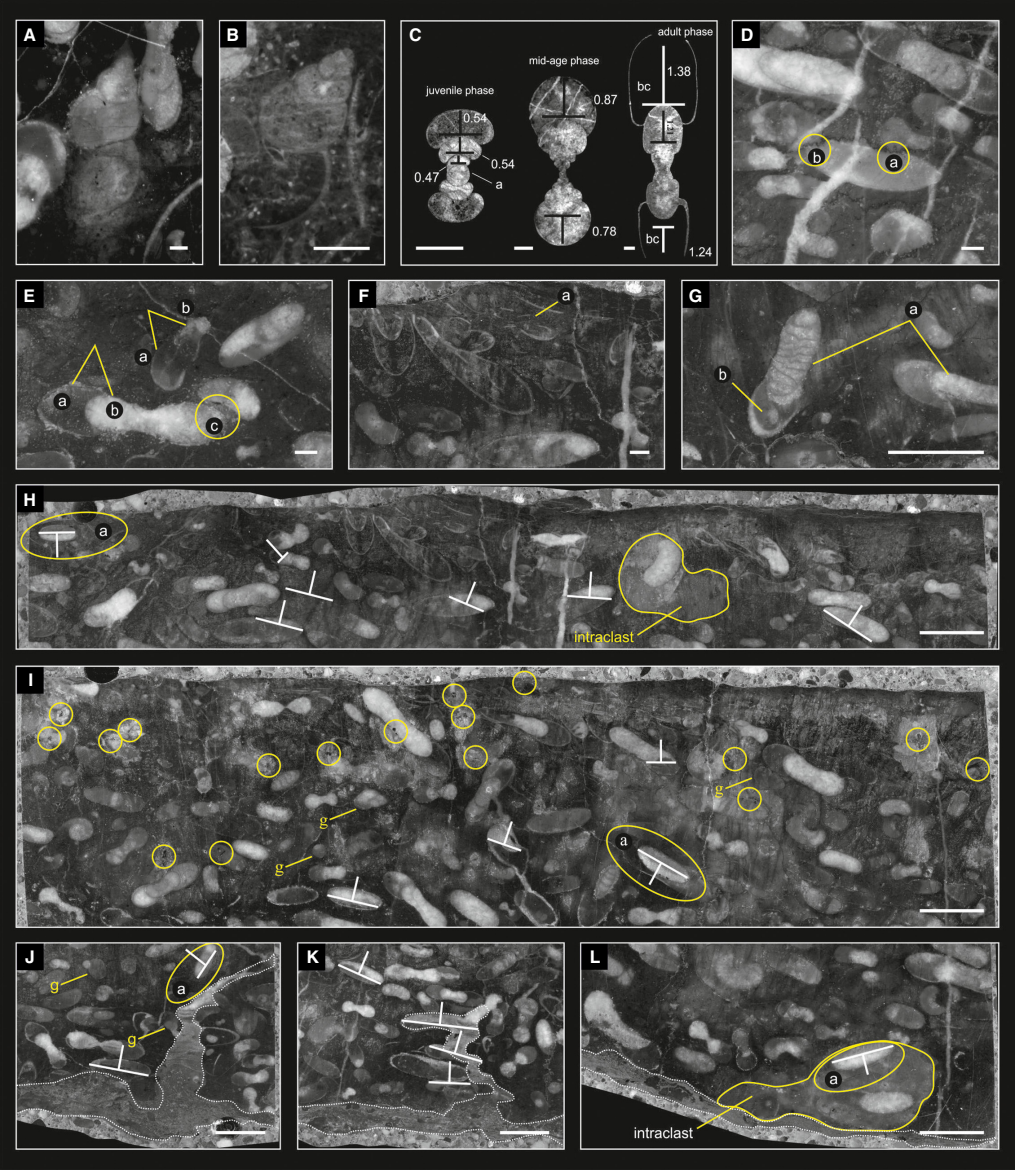
A, B, gastropods within the reference block from section AS I bed AS 6, at slice AS 1C 32 (A) and section AS IV bed C (B). C, transversal thin‐sections of three different ontogenetic phases of *Kasimlarceltites krystyni* with indicated H/W values (juvenile, mid‐age, and adult phase; 2012z0133/0278‐0290). D, E, detailed pictures from the polished slice of bed AS 6/12 representing different textures. D, grain‐ and shell‐supported texture within some parts of AS 6/12, yellow circles point to stylolithic point contact (a) and stylolithic concave‐convex contact (b), respectively. E, matrix‐supported part of AS6/12 with some concave‐convex‐contact (yellow circle). F, fragmented ammonoid shells from the topmost part of slice AS 1C 21. G, ammonoid shells with secondary calcite filling at the phragmocones, and matrix filling the body chamber at slice AS 1C 27. H, I, geopetal fills of ammonoid shells, two specimens (yellow circles) show inverse positions and therefore indicate redeposition after lithification. I, Pyrite crystals resp. framboids (precipitation; small yellow circles). H, AS 1C 21. I, AS 1C 44. J, K, synsedimentary dyke fissure, filled with laminated sediment; most geopetal structures show similar orientation. J, slice AS 1C 14. K, Slice AS 1C 11. L, Slice AS 1C 46 – erosional surface at the bottom; intraclast yielding ammonoids with inverse geopetal fills. White lineations indicate orientation of geopetal fills. Scale bars: A–F, 1 mm, G‐L, 10 mm.

Ninety‐four to 99% of the shells found within the mass occurrence of bed AS 6 at the section Aşağiyaylabel I (AS I) are identified as the ammonoid species *Kasimlarceltites krystyni*. The rest consists of the ammonoids *Sirenites senticosus* and *Klipsteinia disciformis* along with *Omphaloptycha*‐like gastropods (Fig. 8A, B). Furthermore, the ammonoid coquina is covered by halobiid bivalves (for taxonomic details see Lukeneder & Lukeneder 2014). The fact that nearly 99% of the shells belong to *K. krystyni* and the rare additional ammonoid and gastropod species document a paucispecific assemblage (Kidwell *et al*. 1986). The macrofauna of the ammonoid mass occurrence of the sections AS IV, KA I and KA II shows nearly the same composition as found at AS I. Besides the dominance of *Kasimlarceltites*, some *Omphaloptycha*‐like gastropods and thin‐shelled halobiid bivalves as well as fragments of corals and sponges are also present. AS IV additionally contains siliceous sponge spicules (Fig. 7B). Different from AS I, AS IV and KA II, *Kasimlarceltites krystyni* is less dominant in section KA I, where it represents only 50% of the ammonoid specimens (Fig. 7E).

### Number of ammonoids and gastropods

An extrapolation of the counted ammonoids (3,253) and gastropods (211) from the reference block (section AS I, bed AS 6) for the whole extension of the *Kasimlarceltites* bed detected to date (AS I, AS IV, KA I, KA II; 5 km^2^) gives nearly 775 million ammonoids and 50 million gastropods. These values were extrapolated using the following calculation:

$$\begin{array}{c} x\;=\;\frac{\mathrm{no}.\;\mathrm{of}\;\mathrm{specimens}\;\mathrm{of}\;\mathrm{one}\;\mathrm{group}\;\mathrm{from}\;\mathrm{the}\;\mathrm{reference}\;\mathrm{block}}{\mathrm{square}\;\mathrm{footage}\;\mathrm{of}\;\mathrm{the}\;\mathrm{reference}\;\mathrm{block}}\cdot \;\mathrm{square}\;\mathrm{footage}\;\mathrm{of}\;\mathrm{the}\;\mathrm{whole}\;\mathrm{extenion} \end{array}$$

#### 3D‐arrangement of ammonoid shells

The orientation of the ammonoids was analysed to determine their distribution (random or uniform). For this purpose we analysed the significant orientation of the aperture as well as the orientation of an imaginary lineation and plane through each ammonoid of the reference block from bed AS 6 of section AS I (Fig. 5A).

Aperture orientation (Fig. 6A) shows a bimodal NNW/SSE distribution with a preferred orientation of 47%. Although the vector mean of 156° is SSE directed, the maximum class volume (15.1%) is located within the interval 320°–340° and is therefore NNW directed. Analysing the dip in combination with the dip direction of the imaginary lineation (Fig. 5A, C, D) within a stereographic plot (Fig. 6B) revealed a preferred (46.8%) SE orientation of the lineations (100% would indicate a perfect parallel orientation). The lineations (Fig. 6B) show a slight inclination of 18° toward SE (= 144°).

Analysing ammonoid orientation with respect to the dip and azimuth of the imaginary sagittal‐plane through each ammonoid (Fig. 5A, E, F), we found that 66.2% (preferred orientation) are inclined with 14° toward 155° (SSE; Fig. 6C, D). The true center of gravity (cone of confidence; green circle), comparable within the mean of the linear statistic, plots with a significance of 0.05 at 155°/14° (Fig. 6D).

### 3D‐arrangement of gastropod shells

Analysing the aperture direction of the gastropods within a rose diagram (Fig. 6E) showed a bimodal SSE/NNW distribution with a preferred orientation of 88%. The maximum class volume (30.5%) lies at the interval 140°–160°, at which the vector mean plots SSE‐directed at 156°. Dip and dip direction of the lineations (A:B) of the gastropods furthermore show a preferred SSE orientation (72.4%), with an inclination of 24° toward 153° (Fig. 6F).

### Shell morphology

The genus *Kasimlarceltites* is morphologically (i.e. serpenticone, highly evolute; see Lukeneder & Lukeneder 2014) similar with, and in this case taxonomically closely related to other celtitid ammonoids such as *Aplococeras*,* Lecanites* and *Celtites*. The *Kasimlarceltites* shell morphology shows a trend from spherical (embryonic stage) via strongly depressed (juvenile phase) to compressed forms (adult to pre‐adult stages; Fig. 8C). This is also reflected in WER (whorl expansion rate) and W/D (whorl width/diameter) measurements (Lukeneder & Lukeneder 2014). Whilst most WER values (Korn 2000) of *K. krystyni* range between moderate (1.61–2.0) and moderately high (2.01–2.4), some of them show even high WER values (e.g. 2.6).

### Size, shape and sorting

The size of the virtually segmented planispiral ammonoids (Fig. 4) from the block of bed AS 6 ranges from 1.5 to 27.7 mm. This is quite similar to the size variation of the physically segmented specimens of *Kasimlarceltites krystyni* from bed AS 6 at section AS I, where ammonoid size is 0.6–33.0 mm (Lukeneder & Lukeneder 2014). Within block AS 6, ammonoids are abundant, but rare gastropods were also found (Fig. 8A). The virtually segmented gastropods are helical and their size ranges from 2.8 to 11.3 mm (Fig. 8A). The size of *Kasimlarceltites* at AS IV ranges from 4.4 to 25.9 mm, at KA I from 1.6 to 20.2 mm and at KA II from 3.0 to 26.8 mm. Not only at section AS I bed 6, but also at the other localities, small ammonoid shells (ammonitellae, juveniles or microconchs) are mixed with large shells (adult forms or macroconchs; Fig. 7A–F).

### Biofabric

**Figure 9:**
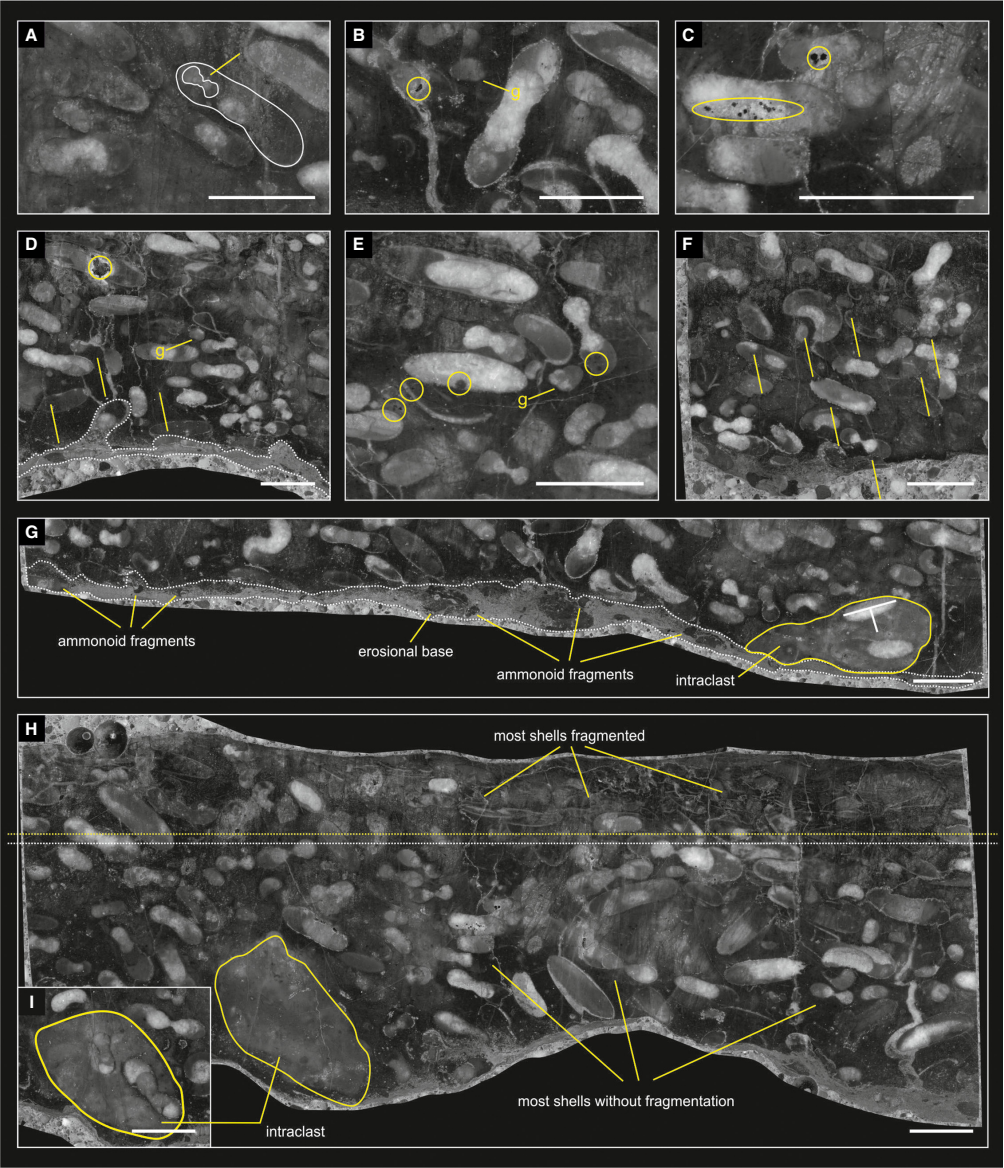
Different slices of the reference block of bed AS 6. A, ammonoid shell containing another ammonoid shell fragment within its body‐chamber (slice AS 1C 37). B–G, Pyrite crystals resp. framboids (precipitation; B–E; AS 1C 47, AS 1C 56, AS 1 C 51, AS 1C 33), dissolution patterns such as stylolithic structures (F; AS 1C 39), and an erosional base (D, AS 1C 51; G, AS 1C 46). H, AS 1C 56 – lowermost two‐thirds of the slice shows mostly complete ammonoids, whereas the topmost third is dominated by mostly fragmented ammonoid shells. H–I, Intraclast yielding ammonoid shells. I, AS 1C 54. All scale bars: 10 mm.

The biofabric differs between beds (Fig. 7A–F). In some places it is matrix‐supported (Fig. 7A, F), which yields scattered ammonoid shell remains. In others it is shell‐supported (Figs 7B–E, 8D), which represents an ammonoid concentration. Interfacial contact zones and areas between the ammonoid shells range from single point‐contacts (Fig. 8Da, yellow circle) to concave/convex contacts (Fig. 8Db, Ec, yellow circles). At all the investigated sections where the *Kasimlarceltites* acme zone was found (AS I, AS IV, KA I and KA II), the sediment alternates between beds bearing *Kasimlarceltites* in masses (shell‐supported structure, as seen at section AS I within bed AS 6; Fig. 9A–H), and beds containing only rare ammonoids with a scattered distribution (matrix‐supported). Even the shell‐supported beds, however, still contain parts, for example at KA II (Fig. 7F) or at AS I (Fig. 7A) that are matrix‐supported.

### Sedimentary infilling

Most ammonoid body chambers are filled with matrix accompanied by shell fragments (Figs 8F–Gb, 9A). Phragmocones are typically filled by sparry calcite (Fig. 8Ga), except for fragmented ones, which also contain matrix (Fig. 8Fa). Most geopetal structures show a similar arrangement in bed AS 6 (Fig. 8H–L, indicated by the base line of the sparry calcite and white arrows normal to them). Nonetheless, a number of ammonoid shells have inverse geopetal fills (Fig. 8Ha–Ia, yellow circles), and some intraclasts yield shells with inclined geopetal structures (Fig. 8La). Inverse geopetal structures are recognisable by sparry calcite at the bottom, in contrast to ‘normal’ calcitic tops, and therefore point to reworking of the ammonoid shells after initial deposition (Fig. 8H–L). Ammonoids in AS IV, KA I and KA II show the same patterns. Most body chambers are filled by background sediment, and phragmocones are crystallized by sparry calcite (Fig. 7B–F). Especially within sections AS IV and KA I (Fig. 7B–E) the calcite filling of the phragmocones preserves the fine structures of the shells, the chambers or of the siphonal tubes (Fig. 7C, yellow circles) and enables them to be measured. The geopetal structures within the mass occurrence at sections AS IV, KA I and KA II reflect almost the same depositional conditions as in AS I (Fig. 8H–L). The inverse geopetal structures in AS IV (Fig. 7C) confirm the overturned nature of the whole succession.

### Encrustation, abrasion and bioerosion

Lukeneder & Mayrhofer (2014) found no traces of encrustation, abrasion or bioerosion in the *Kasimlarceltites* mass occurrence (Fig. 8A–L). This indicates a relatively short exposure time on the sea floor. Precipitation of framboidal pyrite took place after burial. This pyrite growth is visible in Figures 8I (small yellow circles) and 9B–F (small yellow circles). Additionally, Figure 9F shows pressure solution in the form of stylolithic dissolution seams on many ammonoid shells; such shells, however, are recorded only in some areas of the reference block (AS 6). Framboidal pyrite is common at sections AS IV, KA I and KA II too, where it commonly occur within the background sediment and within the sediment‐filled body chambers of the ammonoids (Fig. 7B–F).

### Mode of preservation and fragmentation

Within the thin‐ and polished sections, but also within the 3D‐reconstructions, most ammonoids within the lowermost two‐thirds of bed AS 6 are well preserved; the topmost third of the layer, in contrast, yields mainly crushed shells and shell fragments (Fig. 9H). Most of the ammonoids exhibit the body chamber and lack marks of bioerosion and infestation (e.g. borings). Unfortunately, the ammonoids were hardly separable from the limestone matrix and, specifically, the body chambers could not be saved during physical extraction from the embedding limestone. The same holds true for the gastropods, for which only virtual segmentation could be performed. Nonetheless, due to the great abundance, enough specimens could be extracted for the taxonomic investigation (Lukeneder & Lukeneder 2014). In almost all ammonoids the shell is preserved as recrystallized calcite. Most phragmocones are filled with this secondary calcite too (Fig. 8Eb, Ga), whereas body chambers are filled with sediment (Fig. 8Ea, Gb). Chamber walls and a series of siphuncle tubes are well preserved (Figs 7C, 8G).

### Genetic classification

**Figure 10:**
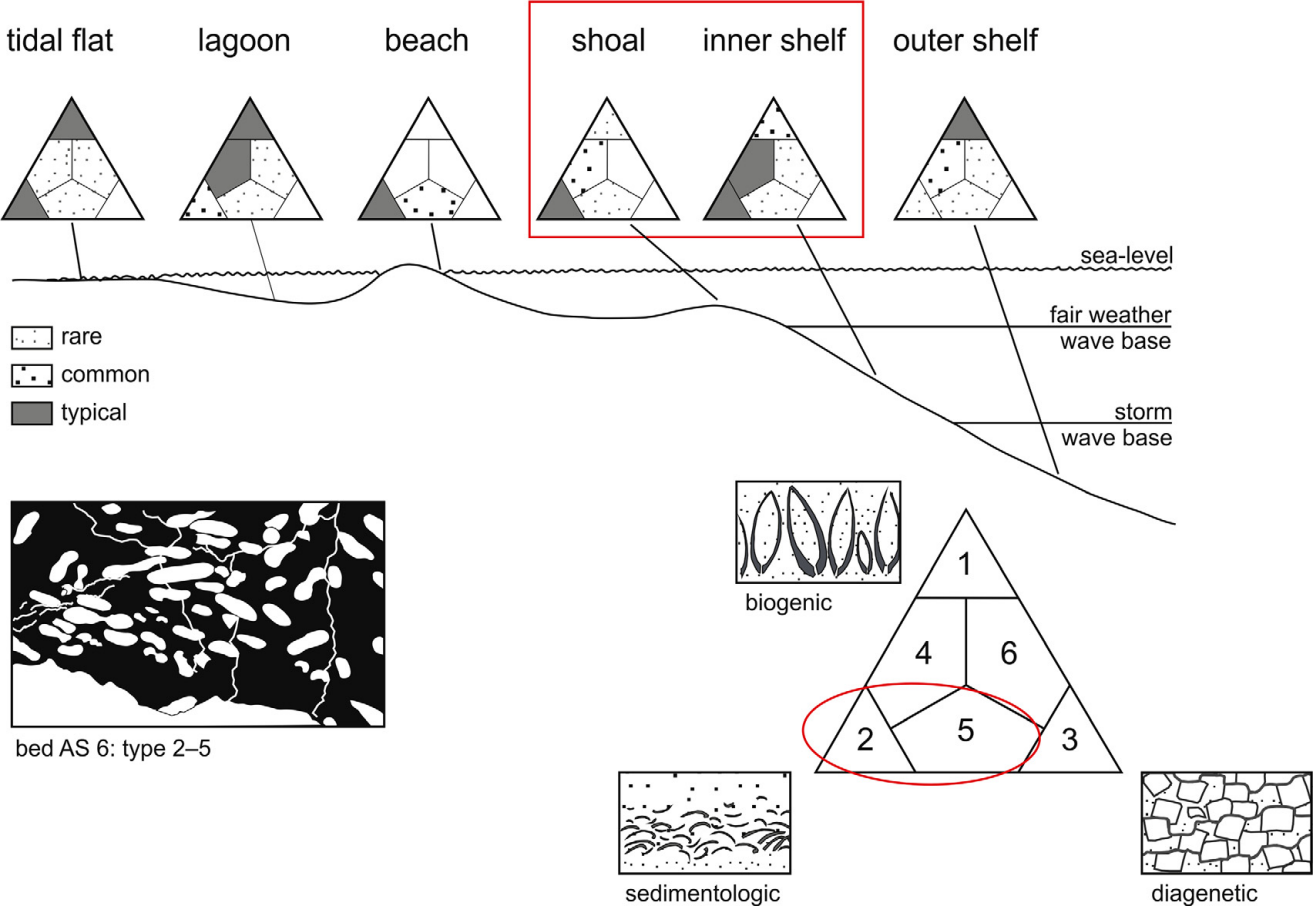
Ternary diagram, adapted from Kidwell *et al*. (1986), of genetic types of skeletal accumulations, with indicated ‘genetic’ position of the *Kasimlarceltites* mass occurrence.

Comparing the *Kasimlarceltites* mass occurrence with the three possible end‐members of the ternary diagram of genetic types of skeletal accumulations (Kidwell *et al*. 1986), a sedimentological genesis is most plausible (Fig. 10). A mixture between a sedimentological end‐member (type 2) and a mixed concentration (type 5; Kidwell *et al*. 1986) is assumed due to the stylolithic structures (e.g. Fig. 9F) and the ammonoid shell alteration from primary aragonite into secondary calcite.

## Discussion

Within this study a quantification of the specimens forming a fossil mass occurrence was done for the first time, which is furthermore one of the most important findings within this study. Besides this quantitative finding, taphonomical and sedimentological interpretations shed light into the genesis of this mass occurrence. Different hypotheses for the origin of such shell concentrations have been proposed in the last decades, from ‘primary biogenic concentrations’ (Fürsich & Oschmann 1993), over ‘catastrophic mass mortality accumulations’ and ‘post‐spawning mortality accumulations’ (Doyle & MacDonald 1993) to ‘winnowed, condensed or resedimented accumulations’ (Doyle & MacDonald 1993; Fürsich & Oschmann 1993).

In studying the genesis of such mass occurrences, Fürsich & Oschmann (1993) defined nine types of skeletal concentrations: ‘fair‐weather wave concentrations’, ‘storm wave concentrations’, ‘proximal tempestites’, ‘distal tempestites’, ‘current concentrations’, ‘primary biogenic concentrations’, ‘winnowed concentrations’, ‘transgressive lags’ and ‘condensed concentrations’. Those authors furthermore described how the genesis can be identified based on taphonomic studies.

Due to the lack of cross‐bedding within the *Kasimlarceltites* mass occurrence, lack of abrasion and only minimal shell breakage, a genesis of the ammonoid mass occurrences by ‘fair weather waves’ or generally by ‘current actions’ seems highly unlikely. The above mentioned features in combination with the well‐sorted components (*Kasimlarceltites* shells – *Kasimlarceltites* shell fragments – halobiid bivalves) along with minimal bioerosion or encrustation at the *Kasimlarceltites* beds does not indicate a genesis by ‘primary biogenic concentrations’, ‘transgressive lags’ as well as ‘condensed’ or ‘winnowed concentrations’. The up to 70% well preserved *Kasimlarceltites* shells do not hint to ‘winnowed concentrations’ or ‘transgressive lags’. ‘Winnowed concentrations’ do not play a role as the fine material is not removed and the shell pavements are much thicker than 1 cm (Fürsich & Oschmann 1993). A mixture of different age fossils or the alteration of intensively burrowed beds, which also would be a hint to ‘winnowed concentrations’ (Brett & Baird 1986; Flügel 2004), are furthermore not observed at the *Kasimlarceltites* shell beds. Additionally, the amount of encrustation, trace fossils or partly dissolved shells is too low (Flügel 2004). The paucispecific character of the *Kasimlarceltites* mass occurrence additionally excludes it from being ‘current’, or ‘condensed concentrations’. Following Fürsich & Oschmann (1993), the paucispecific character points to a genesis of the shell accumulation beds by ‘storm waves’ or ‘tempestites’. Additional hints in the same direction are the good preservation of the ammonoids (scarce shell fragmentation, little to no bioerosion, abrasion or encrustation), the close packing, as well as the sharp erosional base of the bed.

The fabric of the *Kasimlarceltites* beds at sections AS I and KA II represents a bioclastic pelagic floatstone which Fürsich & Oschmann (1993) interpret as deposited by low‐energy transport. The more grain‐supported bioclastic packstone beds at sections AS IV and KA I are, after Fürsich & Oschmann (1993), short‐term higher energy transport deposits.

### Transport mechanisms deduced from spatial fossil orientation

Although most results between the segmented ammonoids from the entire block (*n* ≥ 3000) and those done on fewer ammonoids (*n* = 675; Lukeneder *et al*. 2014) are quite similar (e.g. bimodal NNW/SSE orientation, slightly different vector mean of 156° instead of 150°, Fig. 6A), the preferred orientation (47%) tested within this study is slightly lower. Furthermore, the maximal class volume plots with 15.1% (versus 16.1%) at 320°–340° and therefore exactly opposite to the maximum class volume tested within the smaller ammonoid sample of Lukeneder *et al*. 2014 (tested aperture directions in Lukeneder *et al*. 2014: *n* = 44).

In contrast to these differences, similar results as in the earlier study were obtained for the dip and dip direction of the lineations A:B (18°/144°, 46.8% preferred orientation; Fig. 6B) and for the dip and azimuth of the planes A‐B‐C and their plane poles (14°/155°, 66.2% preferred orientation; Fig. 6C, D). Lukeneder *et al*. (2014) reported 12°/153° as dip and dip direction of the lineation with a preferred orientation of 57.3% (*n* = 74); tested dip and azimuth of the plane was 12°/159° with 59.4% preferred orientation (*n* = 363).

These differences show that a certain sample size is required for stable results. The large difference in the maximum class volume of the gastropods can be explained by the fact that the tested gastropods (only those intersected at least twice) had a much smaller sample size (*n* = 20) which apparently did not reach the sample size required for significant results. Interestingly, the general bimodal orientation is the same (SSE/NNW) as for the ammonoids, but the maximum class volume plots at the 140°–160° class (Fig. 6E–F), i.e. exactly opposite to the maximum class volume of the ammonoids (320°–340°). Accordingly, a different (aperture) orientation behavior of gastropods and ammonoids can be assumed. Despite the small sample size (20 gastropods), we believe the results to be reliable. Interestingly, the aperture direction of the ammonoids in Lukeneder *et al*. (2014; *n* = 44) is the same as that tested for the gastropods (*n* = 20) within the present study.

In summary, all tested apertures show a general bimodal SSE/NNW resp. NNW/SSE orientation. The different orientations of geopetal fills within the ammonoid mass occurrence and the presence of intraclasts containing ammonoids and inverse geopetal structures (Figs 8L, 9H, I) hint to a reworking of the fossils after primary deposition. At the same time, the absence of encrustation, abrasion or bioerosion argues against a lengthy exposure time of the shells on the seafloor. A possible explanation for the readjustment of the geopetal structures is quick burial, reworking shortly after lithification, and transport with the still unconsolidated surrounding sediment to final deposition. The general SSE imbrication of the ammonoids within the stereographic analyses also hints to a slight but significant sediment movement or current direction toward NNW (see Lukeneder *et al*. 2014). As the carbonate platform of the Dipoyraz Dag is located E of Aşağiyaylabel, and the Kartoz Fm. generally shows a N–S striking orientation, a westerly transport would have been more logical. As the wider Isparta Angle area (an area around Isparta bearing a prominent tectonic feature, the Isparta Angle; Altuncu *et al*. 2007) shows a strong post‐sedimentary tectonic compressional overprint (Robertson 2000), local rotations could have contributed to the interpreted NNW movement. Anyhow, an additional possibility might be represented by currents which ran parallel to the slope resp. the platform edge.

Krumbein (1939), Potter & Pettijohn (1977), Rust (1972) and, more recently, Millane *et al*. (2006) have argued that pebble orientation in alluvial sediments might indicate the flow direction. Nagle (1967) and Brenchley & Newall (1970) reported that freely moving conical shells generally are orientated parallel to unidirectional currents (apex up‐current). Potter & Pettijohn (1977) analysed the orientation of discs by gravity and by gravity‐plus‐current action. They concluded that a shifted maximum concentration within a stereographic plot points to down‐current transport of these discs (see also Lukeneder *et al*. 2014). The orientation data of the planispiral cephalopod shells thus substantiate the supposed transport.

Although we found no internal size sorting of the ammonoid‐shells itself, we discovered that the sediment within the accumulation beds is graded from well preserved ammonoid shells at the bottom of the bed (lower two‐thirds of the bed), over fragmented ammonoid shells at the upper third of the bed, to thin‐shelled halobiids which cover the beds. This represents a size grading of the components within the *Kasimlarceltites* beds. This kind of grading, in combination with the fact that the *Kasimlarceltites* acme zone exists of several shell beds resp. event beds, intercalated with the autochthonous background sediment, hints to a genesis by gravity flows (Flügel 2004). The gravity flows were most probably triggered by earth quakes, as we already know that during this time the Isparta Angle area was tectonically active. As the accumulation beds show more or less the same thickness and packing density along the whole extension, the same history of development can be assumed. In contrast to this, different thicknesses of the whole acme zone, which ranges between 1.8 m at AS I and 16.5 m at KA II, would argue for different conditions concerning its preservation.

### Genetic classification of the *Kasimlarceltites* mass occurrence

According to Kidwell *et al*. (1986), fossil concentrations are generally not solely formed as biogenic concentrations, sedimentological concentrations or diagenetic concentrations. They are typically a mixture of two or perhaps of all three concentration types (Fig. 10). In the *Kasimlarceltites* mass occurrence, the sedimentological genesis dominates. A late diagenetic influence is evident from pressure solution remnants along stylolithic structures. Classifying the *Kasimlarceltites* mass occurrence in the ternary diagrams of Kidwell *et al*. (1986) yields type 2, influenced by type 5 (Fig. 10). This would mean deposition between a beach or an inner shelf environment (Fig. 10; Kidwell *et al*. 1986). Concerning the studied microfacies, the inner shelf environment would be more probable. Sedimentological concentrations refer to ancient hydraulic processes (i.e. hardpart concentration), whereas diagenetic concentrations refer to physical and chemical processes (e.g. compaction, selective pressure solution, destruction of hard parts in adjacent beds; Johnson 1960). This also points to primary hydraulic processes in combination with subordinate physical and/or chemical processes (stylolites) for the genesis of the *Kasimlarceltites* mass occurrence.

The most plausible scenario for the genesis of the shell‐supported *Kasimlarceltites krystyni* mass occurrence is short‐term high‐energy events such as gravity flows (proximal tempestites or maybe turbidites).

### The *Kasimlarceltites* mass accumulation as a fossil‐Lagerstätte

Seilacher (1990) classified fossil‐Lagerstätten into two major groups: conservation and concentration deposits. Since fossils within the concentration type are preserved in high quantity, one might tend to interpret the *Kasimlarceltites* case as a concentration‐Lagerstätte, possibly in form of a condensation deposit (Seilacher 1990). The ammonoids, however, are preserved not only in high quantity, but also in good quality. Besides adult apertures, delicate shell structures such as ribs and growth lines, chamber‐ and suture details, and even some siphuncle tubes are well preserved. The presence of all shell size classes from juvenile to adult specimens is also remarkable. A possible explanation for the genesis of the primary accumulation deposit is oxygen fluctuations (Hemleben 1976; Wendt 1976, 1995; Seilacher 1990) with short‐term anoxic conditions in the water column, which should have led to mass mortality of the ammonoids. The pyrite concentration and dark colour of the limestone beds argue for recurrent short‐term anaerobic conditions. As the ammonoids are nektonic organisms and therefore they should have been able to escape anoxic bottom water conditions, the anoxic conditions are interpreted to have been extended throughout the water column, at least for short time. Enhanced tectonism and volcanism, which led to an increase in temperature and with them to an increase in nutrient supply, are plausible reasons for widespread anoxic conditions (Arthur & Sageman 1994; Leckie *et al*. 2002; Meyer & Kump 2008). Another potential explanation is methane degassing triggered by tectonic instabilities. This is planed to be tested by stable isotopes within future research. Either scenario would have led to recurrent ammonoid mass mortality by intensified ecological changes during the Carnian Pluvial Episode. We therefore interpret that the primary mass accumulation was most probably triggered by oxygen fluctuations. Minimal encrustation or bioerosion indicate lack of bottom dwellers and/or rapid burial. Reworking and transport by gravity or turbidity flows led in a second step to the present concentration‐Lagerstätte type *sensu* Seilacher (1990).

### Ecology and primary habitat of *Kasimlarceltites*

As the *Kasimlarceltites krystyni* mass accumulations subsequently experienced transport and therefore are allochthonous, the habitat and mode of life of *Kasimlarceltites* cannot be deduced from surrounding sediments. Since *K. krystyni* is currently known only from Aşağiyaylabel, any interpretations of its mode of life can only be made based on related taxa whose habitat has already been established within an autochthonous context.

Numerous studies deal with the relationship between ammonoid shell morphology and habitat (e.g. Ziegler 1963, 1967; Kauffmann 1977; Donovan 1985; Batt 1989, 1993; Jacobs *et al*. 1994; Westermann 1996; Neige *et al*. 1997; Navarro *et al*. 2005). For example, conch parameters such as shell morphology or siphuncle strength can be indicators for specific primary habitat conditions (e.g. depth, temperature; Westermann 1996; Klug 2002; Ritterbush & Bottjer 2012; Ritterbush *et al*. 2014).

Therefore the reconstructed modes of life of the genera *Aplococeras*,* Lecanites* and *Celtites* (celtitid ammonoids; i.e. serpenticone, highly evolute, ‘primitive’ ceratitid suture) can yield an approximation for the mode of life and habitat of *Kasimlarceltites*. The above‐mentioned genera have been interpreted by Assereto (1969) from the Latemar or Marmolada carbonate platforms (Dolomites, Italy) as shallow ‘platform faunal associations’. Vörös (2002) in contrast described *Aplococeras* as related to peri‐platform environments. According to Assereto (1969), celtitids are rare to absent in deeper basinal environments. The facies dependency was interpreted to be controlled by ecological factors (Brack & Rieber 1993, 1996). These assumptions on a facies or depth gradient dependency of celtitids, hence also of *K. krystyni*, would fit well the interpretation of the Carbonate member Unit A at Aşağiyaylabel. The base of the Carbonate member is, based on its microfacies (e.g. bioclastic wackestones resp. floatstones) and faunal components (e.g. transported ammonoids, sponges, gastropods), interpreted as deposited within a shelf or deeper ramp (Lukeneder *et al*. 2012). For information on facies dependency of Mesozoic ammonoids see Westermann (1996) and Lukeneder (2015). Manfrin *et al*. (2005) also found celtitid ammonoids in adjacent basinal series surrounding the Latemar platform but reported a faunal mixing with benthic gastropods, and therefore interpreted these findings as clearly transported via storm processes. This fits well with the *Kasimlarceltites* mass occurrence at Aşağiyaylabel. The use of celtitid‐dominated ammonoid assemblages as primary habitat indicators for platform or shallow ramp environments is arguable but needs confirmation through additional shallow‐water ammonoid studies.

The hypothesis that similar shell morphologies can be related to similar habitats or life‐styles prompted us to use the habitat knowledge of forms closely resembling the serpenticone morphotype of *Kasimlarceltites* (e.g. *Paraceltites*,* Psilorceras* and *Celtites*) to help determine the habitat and life‐style of *Kasimlarceltites*. The interpreted habitats of the mentioned genera – *Paraceltites* (Permian; Spinosa *et al*. 1975), *Psiloceras* (Jurassic; Westermann 1996), and *Celtites* (Triassic; Rieber 1973, 1975) – are summarized in Westermann (1996) and evaluated in Lukeneder (2015). Spinosa *et al*. (1975), for example, proposed a ‘tropical platform limestone depositional area’ for the Permian genus *Paraceltites*.

A planktonic life‐style was assumed for costate, celtitid morphotypes (Rieber 1973, 1975; Westermann 1996). As postulated by Korn & Klug (2007), ontogenetic changes in morphology reflect, at least in early ammonoid taxa, a change in the mode of life. Nevertheless, the quality of the swimming capability in ammonoids is somewhat speculative and requires more data. Within *K. krystyni* the early ontogenetic changes from spherical to compressed shell morphologies probably represent a change from a planktonic to a nektonic or active swimming form.

## Conclusions

Comparing the palaeontology, taphonomy, sedimentology and palaeoecology of bed AS 6 (section AS I) with shell beds of the *Kasimlarceltites* acme zone from surrounding sections (AS IV, KA I–II) yields the following conclusions about the genesis of the *Kasimlarceltites krystyni* mass occurrence within the Taurus Mountains of Turkey:

1. More than 3000 ammonoids and 200 gastropods were counted within the 150 × 45 × 140 mm reference block. This yielded an extrapolated number of nearly 775 million ammonoids and 50 million gastropods within the known extension of the *Kasimlarceltites* mass occurrence of 5 km^2^.
2. The ammonoid concentrations are paucispecific; 94–99% of the fossils belong to the ammonoid species *Kasimlarceltites krystyni* in co‐occurrence with rare *Sirenites senticosus* and *Anasirenites crassicrenulatus*. Anyhow, section KA I shows, in contrast to sections AS I, AS IV and KA II, a lower dominance (about 50%) of the genus *Kasimlarceltites*.
3. Orientation measurements performed on the ammonoids prove, as already indicated by Lukeneder *et al*. (2014), that they were transported toward NNW.
4. Sedimentological structures and orientation measurements of the shell‐supported beds of the *Kasimlarceltites* mass occurrence support their interpretation as event beds caused by gravity flows (e.g. debris flows, turbidites or tempestites). Interbedded matrix‐supported sediments contain *Kasimlarceltites* only as scattered elements. Distinct taphonomic features such as preferred alignment and tilted geopetal fills corroborate this interpretation.
5. Supposing a life habitat for *Kasimlarceltites* similar to that of the morphologically and/or taxonomically related genera *Aplococeras*,* Lecanites* and *Celtites* (celtitid ammonoids; i.e. serpenticone, highly evolute), *K. krystyni* may have lived in a shallow platform environment or a peri‐platform environment. A tropical carbonate platform environment can also be inferred when considering the morphological similarities of *K. krystyni* with other serpenticone morphotypes such as the Permian *Paraceltites*, the Triassic *Celtites* and the Jurassic *Psiloceras*.
6. If *K. krystyni* changed its mode of life from planktonic to nektonic, it might also have changed its habitat during ontogeny.

By testing taphonomic, sedimentological, lithological as well as palaeoecological features, we conclude that the present ammonoid mass concentrations at Aşağiyaylabel were initially formed by short anoxic events and secondarily transported by gravity flows (e.g. debris flows, turbidites or tempestites) to their present position. Their primary life habitat might have been a shallow platform environment (Assereto 1969), a peri‐platform environment (Vörös 2002) or a tropical carbonate environment (Spinosa *et al*. 1975). The postulated approximately 9° palaeolatitude for the area around Aşağiyaylabel (Lukeneder *et al*. 2012) also suggests a tropical carbonate‐rich environment for *Kasimlarceltites*.

The *Kasimlarceltites* mass occurrence was deposited during the Carnian Crisis = Carnian Pluvial Episode, which is known for an at least Tethyan‐wide carbonate platform demise caused by changing climate conditions. A warmer and more humid climate paired with hydrodynamic (storms) or seismic events (e.g. earthquakes) may also be seen as a trigger of the ammonoid event beds (through gravity flows such as turbidites, debris flows or tempestites). We underline the dependency between such events and ammonoid mass accumulations.
